# Fifth Annual Meeting

**Published:** 1920-06-26

**Authors:** 


### THE COLLEGE OF NURSING, LIMITED.

### Fifth Annual Meeting.

Under the chairmanship of the Hon. Sir Arthur Stanley, G.B.E., C.B. (Chairman of the Council of the College of Nursing), the fifth annual meeting was held on Thursday, June 17, in the Barnes Hall of the Royal Society of Medicine. It was filled to its uttermost, members having travelled from all parts of the country to be present. Amongst those supporting the Chairman on the platform were the following members of the Council: Sir Cooper Perry, M.D., Miss M. E. Sparshott, C.B.E., R.R.C., Miss Edmondson, R.R.C., Miss M. Wiles, Mrs. A. Cecil Carter, A.R.R.C., Miss Cox-Davies, R.R.C., Miss E. Barton, R.R.C., Miss A. Hughes, Mr. Comyns Berkeley, M.Ch., M.D., Dame Sidney Browne, G.B.E., R.R.C., and the Secretary, Miss M. E. Rundle, R.R.C.

The audience included the following: Miss C. E. Vincent (Leicester), Miss M. E. Sutcliffe (Derby), Miss C. Hoadley (Nurses' Co-operation), Miss C. A. Pike (Secretary, Scottish Board), Miss Cathcart (Edinburgh), Miss E. Mowat (Whitechapel Infirmary), Miss Cable (Salisbury), Miss F. B. Mackintosh (Strood), Miss Constance Worsley (Liverpool), Miss B. Chaff (Truro), Miss A. M. Boycott, Miss F. M. Redl, Miss Foyster (Portsmouth), Miss E. F. Saunders (London Hospital), Miss Gertrude Cowlin (Geneva), Miss E. M. Smith (West Didsbury), Miss Amelia Smith (Northampton), Miss E. M. Musson (Birmingham), Miss Pegg (Dundee), Miss K. Jackson (St. Mary's Hospital), Miss Babtie (St. George's Hospital), Miss M. C. Tisdale (Great Ormond Street), Miss I. Swales (Canterbury), Miss Baillie (Bristol), Miss E. S. Innes (Leeds), Miss D. P. Foster (Liskeard), Miss F. Cann (Norwich), Miss G. Copeman (Paddington Infirmary), Miss M. Lloyd (Newport, I.o.W.), Miss Kendall (Northampton), Miss F. G. Nevile (West London Hospital), Miss A. C. Gibson, Miss G. R. Skinner (Reading), Miss B. E. Henry, Miss H. Alsop (Kensington Infirmary), Miss E. E. Cook, Miss A. Rigby, Miss Pote Hunt (Rochester), Miss M. K. Steele (York), Miss A. B. Barter (Manchester), Miss M. I. Burdett, Miss Lightfoot, Miss R. Darbyshire (Simla, India), Miss McIntosh (St. Bartholomew's Hospital), Miss Margaret Cummins (Liverpool), Miss G. Wolseley Lewis (Stoke-on-Trent), Miss Violet Eales, Miss A. Burgess (Crumpsall), Miss M. Girdlestone, Miss A. Thicknesse, Dame Maud McCarthy, Miss M. Freeth, Miss A. Watt (Oxford), Miss L. Binns (Hull), Miss L. W. Wamsley, Miss J. E. Hills (Halifax), Miss M. E. Pinsent (Royal National Orthopædic), Miss Scovell (Swansea), Miss Spackman (National Hospital), Miss Smeeton (Sheffield), Miss Scott (Brighton), Miss M. Rogers (Hammersmith), Miss E. A. Hancox (Sheffield), Miss L. G. Dalton (Victoria Park), Miss L. S. Begg (Putney), Miss J. Lawrence (Star and Garter), Miss A. M. E. Bodley (Selly Oak), Miss M. M. Biggar, Miss M. A. Bompas, Miss A. C. Rastall (Royal Waterloo), Miss H. A. Lawrence (Hong Kong), Miss M. E. Montgomery (Middlesex Hospital), Miss M. K. Coggins (Wigan), and Miss M. A. Marks (Preston).

The proceedings opened by Miss Rundle (Secretary) reading the notice convening the meeting and the Auditors' report.

**The Chairman,** proposing that the report and accounts presented be received and adopted, said: Last year, as I think we all have realised, was one of the very greatest possible importance, both to the College of Nursing and to the nursing profession generally. In the first place, December of 1919 saw the Act for State Registration of Nurses placed on the Statute Book. (Applause.) I need hardly say that, for the College, that was a matter of supreme importance. Very early in the existence of the College we realised and recognised that there was an almost unanimous demand for State Registration, and we therefore employed all the strength of our numbers in the College to attain that object. I think I remember telling you, in the very early days of the College, that if you could get anything like a membership of 20,000 nurses to back up the demand for State Registration, that Registration would follow very shortly. Well, it came even before we got the 20,000. I think the fact that nurses are now recognised by the State is very largely due to the building up of a great Association such as this, one not merely representing isolated nurses, individual nurses, but also representing the combined opinion of the great training-schools in the whole of the country. (Applause.) There is one special feature—I need not go into details of the Registration Bill—which is satisfactory to us. From the very beginning we have always urged that the majority of the nurses' representatives on the Council, or indeed the majority in the Council itself, should be elected by the nurses themselves who are on the State Register. That principle has been conceded, with the result that there is now a State-recognised body, a General Nursing Council, governing the nursing profession, on which nurses themselves on the Register are in the majority. (Applause.) In other words, the nursing profession has come into its own, and it is master—perhaps I ought to say mistress—in its own house.

### Points in the Report.

The Chairman then touched shortly on the work of the Salaries Committee, whose report, he said, although not entirely adopted and only sent out by the Council of the College of Nursing in the form of suggestions, had acted very largely as a standard, and in the revisions of the pay, and especially the hours of work, that have taken place in every profession since the end of the war, has had a very marked influence in improving the conditions, pay, and status of the nurses. The members owed the Salaries Committee a debt of gratitude. The Chairman alluded to the establishment of the College *Bulletin*, which they felt was necessary and an invaluable means of keeping the headquarters of the College and the various centres in touch with individual members. He hoped the badge, too, would come to be fully recognised. In furtherance of the educational work of the College, eight studentships had been established, the examinations for them showing a very high standard. Lady Cowdray had also been very kind in endowing two more studentships for trained nurses to qualify for the appointment of sister-tutor. The Council had tried to help members of the College in many ways. A system had been instituted under which grants are given to help members to take special midwifery courses in order to qualify for certain Ministry of Health appointments. There are also many occasions for some temporary assistance to enable members to take up some particular post, or to follow some particular branch of the profession. For this purpose the Council had instituted a Loan Fund, from which members who were in need could obtain a loan on easy terms. The College had also its Parliamentary Committee, which is in close touch with all matters relating to nursing that may come before Parliament. The Chairman felt the members could be perfectly certain that,

**The College of Nursing, Limited**—(*continued*).

with this Committee and with the College of Nursing, with its very large membership at its back, the interests of nurses would be well safeguarded in the future.

A Real Haven of Rest.

Another advantage for College members was the opening of the Home of Rest for Nurses at Bonchurch, in the Isle of Wight. The idea had originated with Mrs. Martin Harvey, one of the best friends of nurses. Thanks to her help, and that of the Nation's Fund for Nurses, backed up also by the British Farmers' Fund and the Red Cross, who gave a grant of £4,000, the College had established what Sir Arthur believes will prove to be the first, certainly the model, of Homes for nurses in this country, a real Home, a kind of settlement, in which the nurse can come and go and do what she really likes. The Council had also arranged this year for an insurance policy against illness and accident. Sir Arthur said that in the Royal National Pension Fund nurses had an excellent insurance, and could not get anything better. But something more was needed, something in case of accident or sickness. The Eagle, Star, and British Dominions Insurance Company had given exceptionally favourable terms, and every advantage from those terms will be handed on to the members.

The Nation's Fund for Nurses.

The Nation's Fund for Nurses was established in order to meet the needs, which were two. One was the Endowment Fund for the College, the other was the Tribute Fund—a fund out of which provision could be made for those nurses who, through sickness, or accident, or old age, or any other cause, were unable to follow their profession. The Endowment Fund aimed at £100,000, and thanks to very generous subscriptions, a sum not far short of the £100,000 had been reached. However much they might get for the Tribute Fund, there would for the next few years be ample scope for the use of the money in relieving sad cases. He thought the College could congratulate itself on having had a hand in supporting a fund which was already doing such an immense amount of good. To Lady Cowdray, Honorary Treasurer of the British Women's Hospital Fund, who first took up this fund to Lord Burnham, the proprietor of the *Daily Telegraph*, who had pleaded its cause in the columns of his journal, and to Miss May Beeman, who had given her time to it without fee or reward, he voiced the gratitude of all College members.

£30,000 Accumulated in Eight Weeks.

Last year they had, in accumulated funds, £40,421. They had this year reached a figure of £56,783, and since March 31 last that figure had increased by something not much less than £30,000.

He had given the bright side of the picture first. The other side of it was to the effect that the expenditure, that is to say in the income and expenditure account—nothing to do with the capital fund—had exceeded the income by £782. In the old days, before the war, that would have been received with a thrill of horror; now everybody was so accustomed to their expenditure exceeding their income that nobody thought anything about it. There were exceptional circumstances last year that led to that deficit. It was the first time they had printed the register, and that itself cost £780—almost exactly the amount of the deficit. There was much expense in connection with the Parliamentary work and in relation to the Salaries Committee; no less than 75,000 letters went out of No. 7 Henrietta Street last year. Again, the *Bulletin* cost last year £270. There were, however, always likely to be "exceptional circumstances." They had therefore got to increase their income in every way.

The Greatest Sign of Strength.

There was one other matter in this connection. They had founded, during the last two years, centres of the College in various parts of the kingdom, in order that the individual nurse, who could not possibly come to London or take any part in the work at headquarters, could be brought into touch with them here, and feel she was a living member of the College of Nursing. There were now no fewer than twenty-three centres of the College of Nursing throughout the kingdom, and in four of the centres—Edinburgh, Dublin, Brighton, and Newcastle—residential clubs for nurses had been formed. In prospect of having such clubs were Liverpool, London, and Birmingham. It meant a great deal for the College to know that it had living centres in all those parts where nurses could meet one another and discuss nurse politics, and advise headquarters what was the real feeling among nurses throughout the country. Dundee Centre had purchased a house or cottage at Carnoustie, something on the same lines as the Home of Rest at Bonchurch, and there were indications in other directions that additional centres would do the same.

What the Nurses Have Subscribed.

The Chairman then told the meeting that for clubs and centres the nurses themselves had subscribed no less than £18,000; for a Chair of Nursing, £280; for the General Endowment Fund of the College of Nursing, £20,000; for the Tribute Fund, £11,000; for a scholarship Halifax had subscribed £1,200; and Derby is giving £30 a year for midwifery. In all, during the short time the College had been in existence, the nurses themselves have found towards the cost something over £51,000. He likened the College to the ship in Kipling's story, "The Ship that Found Herself," which he advised the members to read. He thought it not a bad simile for what had happened in the nursing profession. There were in the profession all individuals; they had nothing to bring them together, yet they formed then, as they do now, but they did not then know it, a great whole, which has now been recognised by the State. They knew they had only got to express themselves to show what is the view of the nursing profession, and then both Parliament and the public would be only too ready to do anything which the nurses might ask of them.

Gratitude to the College Staff.

The Chairman concluded by expressing, on behalf of himself and of the Council of the College, their deep sense of gratitude to Miss Rundle and to all the staff. (Much applause.) He felt no association had ever been more loyally served than was the College of Nursing by the staff they had been fortunate enough to gather together around them.

The resolution that the report and accounts be presented was seconded by Miss Copeman, and on the Chairman's request for questions, Mrs. G. M. Jones said she wished to propose that after a given date nurses desiring to become members of the College be asked to give an annual subscription, and that existing members be asked voluntarily to contribute an annual sum, in exchange for which they should be entitled to receive quarterly a copy of the *Bulletin*. She knew of no association or society which did not exact a tribute in the way of an annual subscription. Such a subscription would help keep the College going. The registration fee alone could not.

**The College of Nursing, Limited**—(*continued*).

College had done more for nurses to improve their economic condition and get them better conditions of work than any organisation had done in the memory of anyone. If a thing was worth having, it was worth paying for. (Applause.) When the Report of the Committee on Salaries was published, one paper said how was it the College of Nursing was so keen on raising nurses' salaries when they paid their own officials so badly? That was when the post of organising secretary was advertised and £200 a year was offered. Everybody must know that no woman could possibly live on £200 a year.

Miss Burgess seconded the proposal, which the Chairman then put as a resolution, and it was carried unanimously.

### The Payment of an Annual Subscription.

**The Chairman** explained that when they founded the College of Nursing they were quite uncertain as to its future, and did not want to bind members down to pay an annual sum. Now, having achieved a very great measure of success, it seemed only fair that new members who would reap the fruits of the pioneer work should pay a little more for it. Whatever they passed now could not force existing members to pay a yearly fee. But if they chose to give an annual subscription, it would be gratefully received. Any alteration would have to take the form of advising the Council that steps be taken to alter the Articles of Association, in order to allow the payment of an annual subscription. As things were now, the Articles of Association did not allow of it.

**Miss Gane** urged that consideration should be given to the question whether an annual subscription might not mean an extra to nurses, many of whom were still earning only very small salaries. A great reason for having the College was to have large numbers of nurses combined; they wanted as many as they could get.

**Mrs. G. M. Jones** asked : What had the other associations done for nurses? They had not done anything to compare with what the College had done in the four years since it was started. The College had worked hard for them.

**Miss Pringle** (Cheltenham) suggested the compounding method, so that nurses who wished could give a lump sum. Some did not like to be bothered every year for a subscription.

**The Chairman** pointed out that they could not alter their Articles without a special meeting, and after that a further confirmatory meeting of members, so that this matter would have to be thoroughly threshed out and considered. He put this resolution : That the Council be asked to make such necessary alterations in the Articles of Association as will make it possible for members in future to pay an annual subscription.

The resolution was carried, with three dissentients.

**Miss Biggar** then proposed, and **Miss Worsley** seconded, the re-election of Messrs. Barton, Mayhew and Co. as auditors to the College.

### Thanks to the Council.

**Miss Scovell** (Swansea) proposed a resolution of thanks to the Council for the enormous amount of work and vitality it had put into the College affairs.

**Miss Chaff** (Truro), in seconding, bore testimony to the fact that in the provincial hospitals, the smaller hospitals at any rate down West, the nurses, from probationers upwards, were beginning to realise the great benefit the College is.

### The New Members of the Council.

**Dame Sidney Browne,** G.B.E., R.R.C., took the chair, and read the names of the new Council members, in the order in which they were elected :

*English and Welsh Section.*

The Hon. Sir Arthur Stanley, G.B.E. (Loud applause.)
Miss M. E. Sparshott, R.R.C.
Miss Lloyd Still, R.R.C.
Dr. Jane Walker.
Mr. Russell Howard, F.R.C.S.
Right Hon. Viscount Knutsford.
Miss Elizabeth Mary Wyatt (Queen's Nurse).
Miss Bowden (School Nurse).

*Scottish Section.*

Miss Alice Helen Turnbull, Edinburgh.
Miss Isabel Annie Calder (Ward Sister, Glasgow).

*Irish Section.*

Dr. George Peacocke, M.D.
Miss Annie Michie (Superintendent, Q.V.J.I.N. for Ireland).

**She pointed out that with this election all the Council had now been elected by the nurses themselves.**

**Miss Cox Davies,** as No. 2 on the Register, gave Sir Arthur Stanley the very warmest possible welcome back into the chair. They owed the foundation of the College to Sir Arthur Stanley; he and Dame Sarah Swift were the moving spirits.

**Miss Gibson,** in seconding the vote of thanks, said : The greatest gratitude they could show to Sir Arthur Stanley would be by putting their hearts and minds into this College.

**Sir Arthur Stanley,** resuming his occupancy of the chair, said : He would not have thought, four years ago, it would have been possible to get together a College such as this College of Nursing. It showed a very surprising step forward, a very gratifying step in advance to know they had such a magnificent representative of a hospital, one so worthy of the Council, as Lord Knutsford. He might not always have seen eye to eye with nurses in the past—(laughter)—but the fact that they had been able to assist in his regeneration—(loud laughter and applause)—was one of the most striking successes which the College had yet achieved on the new Council.

The Chairman then paid an eloquent tribute to Dame Sarah Swift. When the history of the College came to be written it would be known that the foundation of the College was due to Dame Sarah. She had retired voluntarily from the Council, because she thought it wanted new blood on it.

**Miss McIntosh** proposed the heartiest thanks to Miss Rundle for all she had done during the last five strenuous years since the College was established. She had always shown unfailing cheerfulness and optimism. Five years of such pioneer work must have been very trying. She had worked splendidly and cheerfully, always keeping one object in view, and she must be very proud to-day to see the result, and the position the College holds in the world to-day. (Loud applause, during which a very fine sheaf of roses was handed to Miss Rundle.)

**Miss Cummings** (Liverpool) seconded the resolution, which was carried by acclamation.

**Miss Rundle,** in replying, said these had been the happiest years of her life. If it had not been for the members nothing would have been done at all, and there would have been no Secretary. She was supported in the office by her staff. There was nothing she could ask them to do which they did not do most willingly.

### The College's Future Home.

**The Chairman** then announced that the Council had every reason to hope that next year the members would have a house of their own, not only for the College, but joined on to it a club as the London centre. (Much applause.) Wild horses would not drag from him where it would be, but part of it, at all events, would be in Cavendish Square, very centrally situated, thanks to the generosity of some of their good friends. (Applause.)

**[The Reports of the College Conferences are unavoidably held over this week.—Ed. "The Hospital."]**

